# Outlier analysis for accelerating clinical discovery: An augmented intelligence framework and a systematic review

**DOI:** 10.1371/journal.pdig.0000515

**Published:** 2024-05-22

**Authors:** Ghayath Janoudi, Mara Uzun (Rada), Deshayne B. Fell, Joel G. Ray, Angel M. Foster, Randy Giffen, Tammy Clifford, Mark C. Walker

**Affiliations:** 1 Clinical Epidemiology Program, Ottawa Hospital Research Institute, Ottawa, Canada; 2 School of Epidemiology and Public Health, University of Ottawa, Ottawa, Canada; 3 Independent Researcher, Ottawa, Canada; 4 Departments of Medicine, Health Policy Management and Evaluation, and Obstetrics and Gynecology, St Michael’s Hospital, University of Toronto, Toronto, Canada; 5 Faculty of Health Sciences, University of Ottawa, Ottawa, Canada; 6 IBM Canada, IBM, Toronto, Canada; 7 Canadian Institute of Health Research, Government of Canada, Ottawa, Canada; 8 International and Global Health Office, University of Ottawa, Ottawa, Canada; 9 Department of Obstetrics and Gynecology, University of Ottawa, Ottawa, Canada; 10 Department of Obstetrics, Gynecology & Newborn Care, The Ottawa Hospital, Ottawa, Canada; 11 BORN Ontario, Children’s Hospital of Eastern Ontario, Ottawa, Canada; University of the Philippines Manila, PHILIPPINES

## Abstract

Clinical discoveries largely depend on dedicated clinicians and scientists to identify and pursue unique and unusual clinical encounters with patients and communicate these through case reports and case series. This process has remained essentially unchanged throughout the history of modern medicine. However, these traditional methods are inefficient, especially considering the modern-day availability of health-related data and the sophistication of computer processing. Outlier analysis has been used in various fields to uncover unique observations, including fraud detection in finance and quality control in manufacturing. We propose that clinical discovery can be formulated as an outlier problem within an augmented intelligence framework to be implemented on any health-related data. Such an augmented intelligence approach would accelerate the identification and pursuit of clinical discoveries, advancing our medical knowledge and uncovering new therapies and management approaches. We define clinical discoveries as contextual outliers measured through an information-based approach and with a novelty-based root cause. Our augmented intelligence framework has five steps: define a patient population with a desired clinical outcome, build a predictive model, identify outliers through appropriate measures, investigate outliers through domain content experts, and generate scientific hypotheses. Recognizing that the field of obstetrics can particularly benefit from this approach, as it is traditionally neglected in commercial research, we conducted a systematic review to explore how outlier analysis is implemented in obstetric research. We identified two obstetrics-related studies that assessed outliers at an aggregate level for purposes outside of clinical discovery. Our findings indicate that using outlier analysis in clinical research in obstetrics and clinical research, in general, requires further development.

## Author summary

We introduce a new way to accelerate clinical discoveries by applying outlier analysis within an augmented intelligence framework. Traditionally, the unique clinical observations that led to breakthroughs like the discovery of Kawasaki disease and treatments for psychological disorders were reported through detailed case reports and case series. However, these methods often miss many such observations due to the intense demands on clinicians’ time and the inefficiency of the case report and case series systems. Our approach reimagines clinical discovery as an outlier problem, where unusual data points within health datasets, identified through outlier analysis, signal important new findings for experts to investigate. We propose a five-step process that begins with defining a patient population and ends with generating scientific hypotheses. This structured approach enhances our capacity to identify novel medical insights and reduces the reliance on happenstance and the subjective selection of what observations to pursue. This framework represents a significant shift towards a more proactive and data-driven method in medical research and ushers in a new era of clinical discovery.

## Introduction

Throughout history, medical knowledge has been advanced through clinical observation [1–3]—the latter also serving as a catalyst for future research and scientific discovery [2–5]. Traditionally, novel clinical observations have been communicated through case reports and case series published in medical journals [6–8]. Examples include the discovery of Kawasaki disease, [9] the discovery of Hantavirus Pulmonary Syndrome, [10] the discovery of the association between statin therapy and rhabdomyolysis, [11] the discovery of disulfiram for managing alcoholism, [12] and the discovery of several treatments for psychiatric conditions [13–15]. Several widespread therapeutics have been discovered through accidental clinical observations; these include aspirin’ anti-thrombotic effects, beta-blockers anti-hypertensive effects, botulism toxin for wrinkle treatments, sildenafil for erectile dysfunction, and glucagon-like peptide-1 for weight loss [16–21]. A common theme across many such discoveries is reporting an observation that stood out against what would otherwise be expected.

Due to their methodological limitations, case reports and case series may have somewhat fallen out of favour in the past two decades [22,23]. Nevertheless, they continue to offer new insights, as was evident by their re-emergence during the COVID-19 pandemic [24–28]. A number of prominent COVID-19 case reports have led the COVID-19 scientific discourse and exploration [29–31]. The role of case reports and case series in communicating new observations, generating hypotheses, and acting as the first step in advancing clinical research remains deeply entrenched in the medical community [32–34]. Even so, unique and valuable clinical observations may remain unreported due to the many competing priorities placed on busy clinicians and the uncertainty of medical journals’ publishable case reports.

Outlier analysis offers a more efficient and streamlined alternative for identifying unique or unusual clinical observations. Specifically, it can identify an unusual observation that does not adhere to an expected behaviour; [35,36] that is, an observation which differs substantially from other observations, leading to suspicions that it originated from a distinct mechanism [37]. In biostatistics, outliers are conventionally considered to be statistical noise and are, therefore, often excluded from analyses [38]. However, distinguishing between statistical noise and an informative outlier and understanding the mechanisms giving rise to the latter may expose important and valuable information. Such an approach is now used in fields outside of medicine, including financial fraud detection, network connection anomalies, malware detection, and quality control in manufacturing processes [35,36]. Within the field of medicine, outlier analysis has been recently reported for the purposes of disease diagnosis, data quality assurance, and medication error screening, as well as for monitoring a patient’s vital signs and then alerting a caregiver when those physiological measures considerably deviate beyond the normal parameters [39–42].

This paper provides a non-technical overview of outlier analysis and considers how clinical discovery may be framed as an outlier analysis problem. Next, a general framework of augmented intelligence is proposed, whereby outlier analysis methods are used to continuously monitor health-related data for novel clinical observations (i.e., deviations), which can then be investigated by content-matter experts. Finally, given that pregnant patients are most excluded from the planning and conduct of pharmaceutical research, [43–45] the use of outliers as means of advancing discoveries in obstetrics can be particularly of value. As such, a systematic review was completed to identify how outlier analysis has been used in obstetric research.

## Definitions and fundamentals of outlier analysis

### Definition of outlier

Healthcare professionals engage in outlier analysis on a daily basis as part of clinical practice. A healthcare professional engaged in diagnosing a patient looks for signs and symptoms not normally observed in healthy individuals. The existence of these signs and symptoms identifies a patient as an “outlier” when compared with the expected healthy presentation; the disease is the underlying mechanism that gave rise to the outlier observation. This intuitive, clinical-based understanding can be transferred from the individual patient-physician interaction to outlier analysis of multidimensional data.

The interest in identifying and addressing outlier observations in a set of data has been ingrained in the practice of statistics in the past century [37]. Conventionally, the aim of identifying outliers has been to eliminate such observations from the analysis (i.e., data cleaning) [37,46,47]. With the significant growth in the fields of statistics and machine learning throughout the past three decades, applications of outlier analysis found their way outside the realm of data cleaning, and new terms emerged that are now frequently used interchangeably. Specifically, the terms *outlier*, *noise*, *anomaly*, and *novelty* appear frequently in this literature. Of these, *anomaly* and *noise* are perhaps the most common terms used to refer to observations that do not align with an expected or predefined behaviour or characteristic.

The most frequently used definition of *outlier* comes from the 1980 book *Identification of Outliers* by D.M. Hawkins: “An outlier is an observation which deviates so much from the other observations as to arouse suspicions that it was generated by a different mechanism.” [37] Similarly, an *anomaly* is frequently defined as an entity that does not conform to a defined notion of the normal [46–48]. In many instances, the terms *outlier* and *anomaly* are used interchangeably [49]. Attempts at differentiating various terms, one from another, are mostly related to the aim of the analysis and the degree to which the detected observation is found to be compelling or interesting: *anomalies* are usually associated with observations that have real-life relevance, [50] *novelties* are associated with observations or patterns that have not been detected before, [51] and *noise* is associated with observations that clearly are due to random error and should be removed or accommodated [52,53]. We will be using the term *outlier* as an all-encompassing term that includes potential anomalies, novelties, and noise. A consistent element in the various published definitions of outliers is a predefined or expected normal behaviour, outcome, or model. In essence, an outlier is defined by its exclusion or deviation from what is understood as normal; and different outlier analysis methods are mostly distinguished by how that normal is defined and how the exclusion of non-normal observations is measured.

### Characteristics of outliers

**Fig 1:**
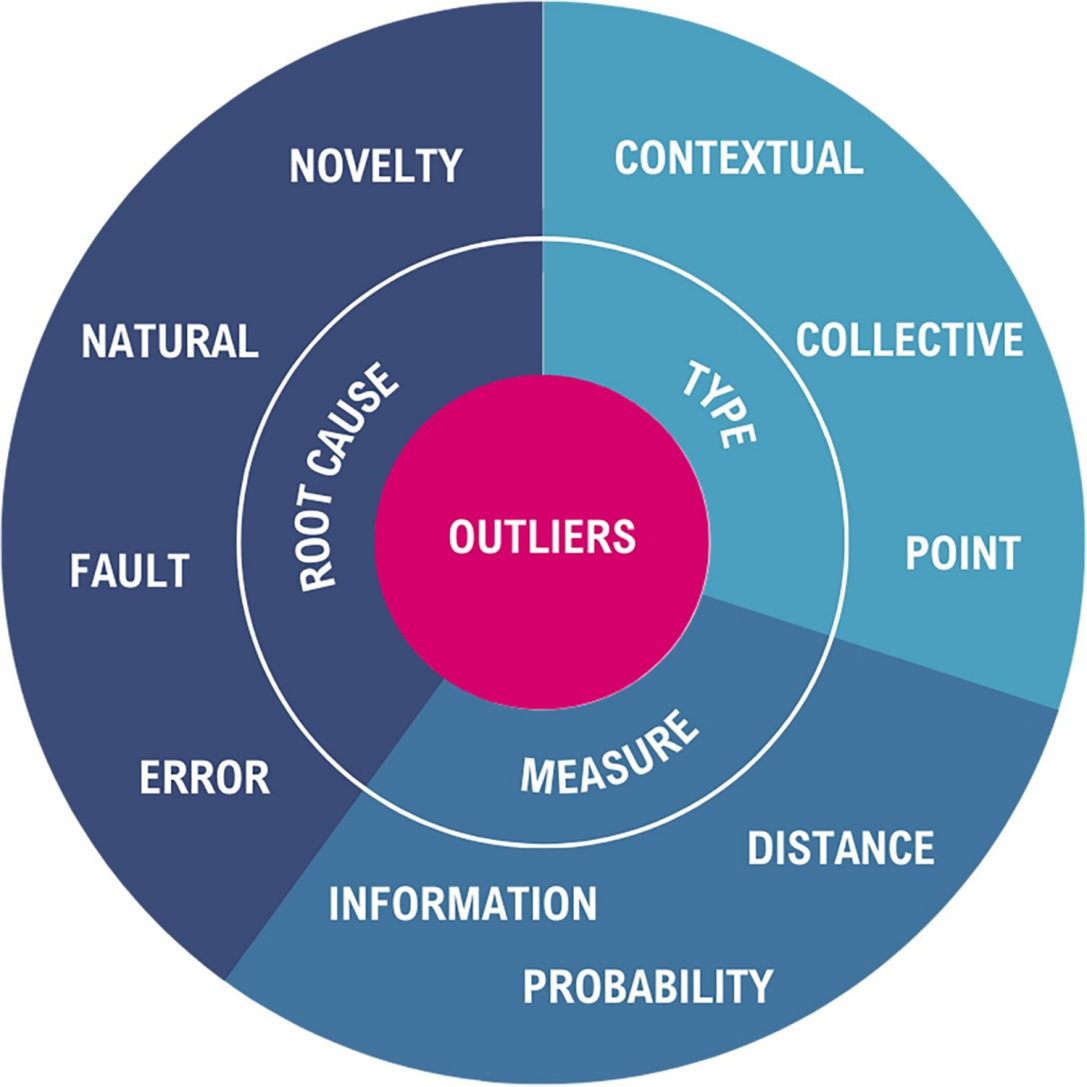
Characteristics and Categories of Outliers.

We can characterize outliers by three attributes: root cause, type, and measure. The reader can find a graphic representation of these characteristics and their categories in Fig 1, followed by subsequent explanations of these attributes. We provide an outline of the characteristics, categories within these characteristics, and clinical examples for each category in Table 1 at the end of this section.

**Table 1:** Clinical Examples of the Various Categories of Each Characteristic of Outlier.

Characteristic	Category of Characteristic	Clinical Example
Root cause	Error	Entry of an additional digit in the weight field in a patient’s electronic record.
	Fault	Congestive heart failure causing shortness of breath in a patient.
	Natural deviation	An exceedingly tall individual in height, with no underlying pathological process.
	Novelty	Exposure to a pharmaceutical compound for an unrelated indication causing an unexpected alteration to the disease being studied.
Type	Point	A patient diagnosed with a disease is a point outlier relative to the larger healthy population.
	Collective	The cluster of a rare form of an infectious disease in a defined geographic area.
	Contextual	Physiological changes in pregnancy would be considered an outlier when compared to the general population but are otherwise normal when understood within the context of pregnancy.
Measure	Distance	The distance (degree) of the measured systolic blood pressure of a patient to an accepted upper limit determines if a patient meets the definition of hypertension.
	Probability	A rare adverse event that arises during the therapeutic management of a condition.
	Information	A suspected case of a disease that presents with a wide range of novel signs and symptoms not previously part of the traditional description of that suspected disease.

#### Root cause

Determining the cause that gave rise to the outlier observation is the ultimate goal of outlier analysis. This determination largely depends on domain knowledge, as well as knowledge of the method used to identify an outlier. Various root causes of outliers have been published in the literature [35,40,54–58]. These generally fall into four categories: error-based, fault-based, natural deviation, and novelty-based. Error-based outliers can arise from both human and instrument errors. Fault-based outliers are instances where the underlying system behaves in a manner that is indicative of a breakdown of an essential function or a malicious external activity; these can include disease states, fraudulent activities, or faulty equipment. Natural deviations include chance-based events, as well as those that can be explained by the underlying modelled process but lie to the extreme of the expected behaviour. Finally, and perhaps the most interesting, are the outliers that arise due to a generative mechanism that has not been accounted for in the expected behaviour or outcome. Such outliers may contain valuable information that can further our understanding and expectations of the issue at hand.

#### Type

Outliers are also distinguished by their type—the nature of the outlier in relation to its size and the surrounding context. We can classify outlier types into three categories: point outlier, collective outlier, and contextual outlier [49,54,55,58,59]. A point outlier is an individual observation (data point) that is determined to be an outlier from other observations; this is the most discussed type of outlier in the literature. For example, a patient diagnosed with a disease is a point outlier from the larger healthy population. The second category is the collective outlier, which refers to a group of data points that, by themselves, are not outliers but, when put together, are determined to be sufficiently different from the majority of other points. A clinically relevant example of a collective outlier is the detection of disease outbreaks, where, at a given time, a single case of a rare disease is not by itself an outlier from the expected behaviour of the disease, but a group of cases are an outlier. The final category is the contextual outlier, that is, outliers that are context-specific. A clinical example of a contextual outlier is a pregnancy-related physiological change and the associated signs and symptoms; should these signs and symptoms be reported outside the context of pregnancy, then it would give rise to a consideration of a disease state, which is an outlier to the expected healthy state of the majority of individuals.

#### Outlier measure

A third important characteristic of an outlier is the type of measure that was used to determine its nature. A near-universal element in outlier analysis is when an unexpected outlier is measured against a predefined normal behaviour, outcome, or model. This type of measure can take several forms. The most common are distance-based measures, probability- and density-based measures, and information-based measures [40,54,55,60–62]. Distance-based measures identify outliers by judging how far they are from a predefined measure of a normal model or parameters. Most clinical laboratory testing uses this approach to highlight abnormal results. Another common clinical example is blood pressure, whereby if a measurement is a certain distance (in mm Hg) from an accepted upper limit (e.g., a systolic 120 mm Hg), then a patient is considered an outlier and is investigated further for hypertension. Probability-based measures also referred to as density-based measures, identify outliers as an observation that is unlikely to exist in the manner in which it was identified. Unexpected outcomes that can occur during the course of treating a patient can be considered outliers under a probability-based measure. An example would be the progression of a bacterial upper respiratory infection to a case of pneumonia despite adequate anti-microbial treatment. Finally, information-based measures identify outliers by the effect their removal or addition has on our ability to form an accurate normal behaviour, outcome, or model that governs the rest of the data. A disease description with classic signs and symptoms is a good example of an information-based measure, whereby patients who present with signs and symptoms outside of a classic description can be classified as outliers. For example, a patient with preeclampsia presenting with loss of sight. The reason here is that if we are to incorporate “out-of-model” signs and symptoms, the disease description would be much more complex informationally and potentially less accurate to the majority of the observations. An information-based measure can also include what traditionally has been referred to as model-minimization or rule-based approaches.

### Approaching outlier analysis methods

Inherent to the definition of an outlier is what we understand and define as normal or expected behaviour, outcome, or model from which the outlier deviates. A data model is a representation of this norm or expectation expressed in a manner that allows the objective assessment of outliers. The approach to structuring such a model will determine the method that the analyst needs to implement. The aim of this section is not to provide a comprehensive description of outlier detection methods, as such reviews can be found elsewhere [40,54,60–66]. Rather, we focus, instead, on providing a non-technical overview of outlier methods that can be applied to multidimensional data. Multidimensional data may include various data types (numeric, ordinal, and categorical)—such as patient records, as opposed to, for example, visual data (e.g., radiographs). Subsequently, we detail a generic process for determining the prerequisites that are essential for a valid outlier analysis.

### Data labels

Data labels refer to whether, in a given dataset, we know which observation is an outlier and which is a normal observation before we begin our analysis [40,54,60–62]. Outlier analysis is inherently a classification problem, where observations are classified as either outlier observations or normal observations. There are three scenarios that we can derive from data labels. These three types of methods all share an implicit assumption that the normal observations far outnumber the outlier observations [40].

#### Supervised outlier analysis methods

This type of outlier analysis can take place when both normal and outlier observations are known—a situation that lends itself well to the use of supervised methods. Ideally, an analyst would develop a predictive model to distinguish between normal versus outlier observations and then apply the model to new observations to determine their class (normal observation or outlier observation). While supervised outlier analysis methods are likely to provide better results than semi-supervised and unsupervised methods, in practice, it is uncommon to come across a dataset that has both outlier and normal observations comparatively labelled. In addition, it is important to use approaches that can address the strong class imbalance, where outlier labels represent a small proportion of the dataset compared to the normal labels [67]. Supervised outlier analysis methods are identical to any regression or classification predictive models and follow the same approaches for feature selection and model tuning.

#### Semi-supervised outlier analysis methods

This is a scenario whereby only the normal observations are labelled. In such an approach, a data model is built that best represents the existing normal observations; new observations are assessed based on how well they can be explained by the developed data model [40,54,60]. Examples of semi-supervised models utilized for outlier analysis include kernel principle component analysis and one class support vector machine [65].

#### Unsupervised outlier analysis methods

Commonly, outlier analysis problems do not have a normal or an outlier label attached to the observation. In these cases, the analyst is forced to use unsupervised outlier analysis methods. Examples of unsupervised models utilized in outlier analysis are isolation forest and local outlier factor [68,69].

#### Expected characteristics of outliers

Domain knowledge plays an essential part in guiding the approach to outlier analysis. Part of that is the prior determination of the desired characteristics of the outlier, as informed by the content-matter expert. Of the three outlier characteristics, two must be designated by the research team prior to conducting the analysis to allow for the determination of the most appropriate method to use. These two outlier characteristics are type (point outlier, collective outlier, and contextual outlier) and outlier measure (distance, probability, and information).

The largest body of literature on outlier analysis deals with the problem of point outliers [40]. These outlier analysis methods address the task of detecting single observations in a given dataset [49,70]. For a task that requires the detection of contextual or collective outliers, careful assessment of the method to be used must take place, as many point outlier methods will not be able to detect collective or contextual outliers [63]. Contextual and collective outliers may require reframing or redefining the task to allow for the utilization of point outliers methods. [49,63]

Determining the outlier measure is a function of both the domain knowledge and the nature of the available data. In the simplest form of a single-dimension (univariate) outlier analysis task, a domain expert should be able to speculate on how an outlier is usually determined through drawing from experience. However, once multidimensional aspects of the observation are introduced, the choice of an outlier measure can be highly dependent on the quality and characteristics of the dataset [71,72].

#### General model assumptions

As is the case with any statistical analysis, any chosen outlier analysis method would have an inherent set of assumptions that, if not met, may lead to poor results. For example, in a common statistical model like linear regression, typical assumptions include linearity, normality, and independence. In the random forest model, assumptions include independence and feature stability. It’s important to choose a model based on our ability to validate its assumptions [35,55,56,71].

## Clinical discovery as an outlier analysis problem

Classically, clinical discoveries were reported as a form of an unusual observation that stood out against that which was expected [13–15]. Considering the similarities of this classic approach to the definition of outliers, we propose to reframe the topic of clinical discovery into an outlier analysis problem. To that end, we suggest that clinical discovery is a contextual outlier, measured through an information-based approach and with a novelty-based root cause. Further, it is likely that this is a problem that falls under the unsupervised outlier analysis category. Following, we provide an argument in support of this reframing proposal to clinical discovery.

### Novelty-based

While the root cause determination of an outlier observation requires careful investigation by the analyst, as well as the content-matter expert, we can assume that in order for an outlier observation to contribute to clinical discovery, it needs to be generated through a mechanism that is not accounted for in the normal or expected behaviour, outcome, or model. While the discovery of an outlier observation due to other processes may be useful, such as the discovery of potential errors, the underlying generative process can still be accounted for by the normal expected behaviour had the error not taken place.

### Contextual

A clinical discovery is a contextual outlier—it requires specific conditions for it to be detected as such, and in the absence of such conditions, the clinical discovery observation is likely to be missed. It is important to consider that the process of scientific discovery, in general, is not a random one. Discoveries require the investigators to actively guide their efforts to address the topics of interest. Similarly, an outlier analysis approach to clinical discovery needs to be defined within the context of the disease or clinical outcome of interest in order to yield relevant results. Conducting outlier analysis on patient data without actively defining the context and conditions in which clinical discovery is sought is unlikely to detect a valid clinical discovery.

### Information-based

While it is likely that both distance-based and probability-based outlier measures can be used in clinical discovery, we believe that an information-based measure best reflects the human-based approach to clinical discovery. An unusual clinical observation can be clinically described in a myriad of ways: through presentation, diagnostics, therapeutics, progression, or outcomes. However, one thing remains constant in all of these descriptions: the inability to reconcile the presentation with the learned model (i.e., the information summary) of the condition. It is thus likely that information-based measures have the potential to be most suitable for the task of outlier analysis in clinical discovery.

### Unsupervised

Inherent to the task of clinical discovery is the lack of labelled observations that would otherwise reflect a case of clinical discovery. Thus, it is not possible to use supervised outlier analysis approaches. In addition, labelling observations as normal or non-discovery requires significant investment and resource allocation, making it unlikely for us to use semi-supervised outlier analysis approaches. Unsupervised outlier analysis methods are, therefore, likely to be commonly used in the field of outlier analysis for clinical discovery.

## An augmented intelligence framework for accelerating clinical discovery through outlier analysis

Augmented intelligence refers to the implementation of machine learning and statistical learning models to enhance the capabilities, knowledge, and decision-making abilities of humans. Augmented intelligence adds a layer of information to enhance human intelligence [73].

Outliers are unusual and infrequent events. Indeed, the likelihood that an outlier is caused by a novel and previously unknown generative mechanism (i.e., a clinical discovery) is exceptionally rare. Thus, when starting an outlier analysis for clinical discovery, there is a need for awareness of the low probability of finding an outlier that might represent clinical discovery. However, even with an expected low probability of capturing a clinical discovery observation, outlier analysis will arguably detect more clinical discovery observations than the classic, human-based approach communicated through case reports and case series.

**Fig 2:**
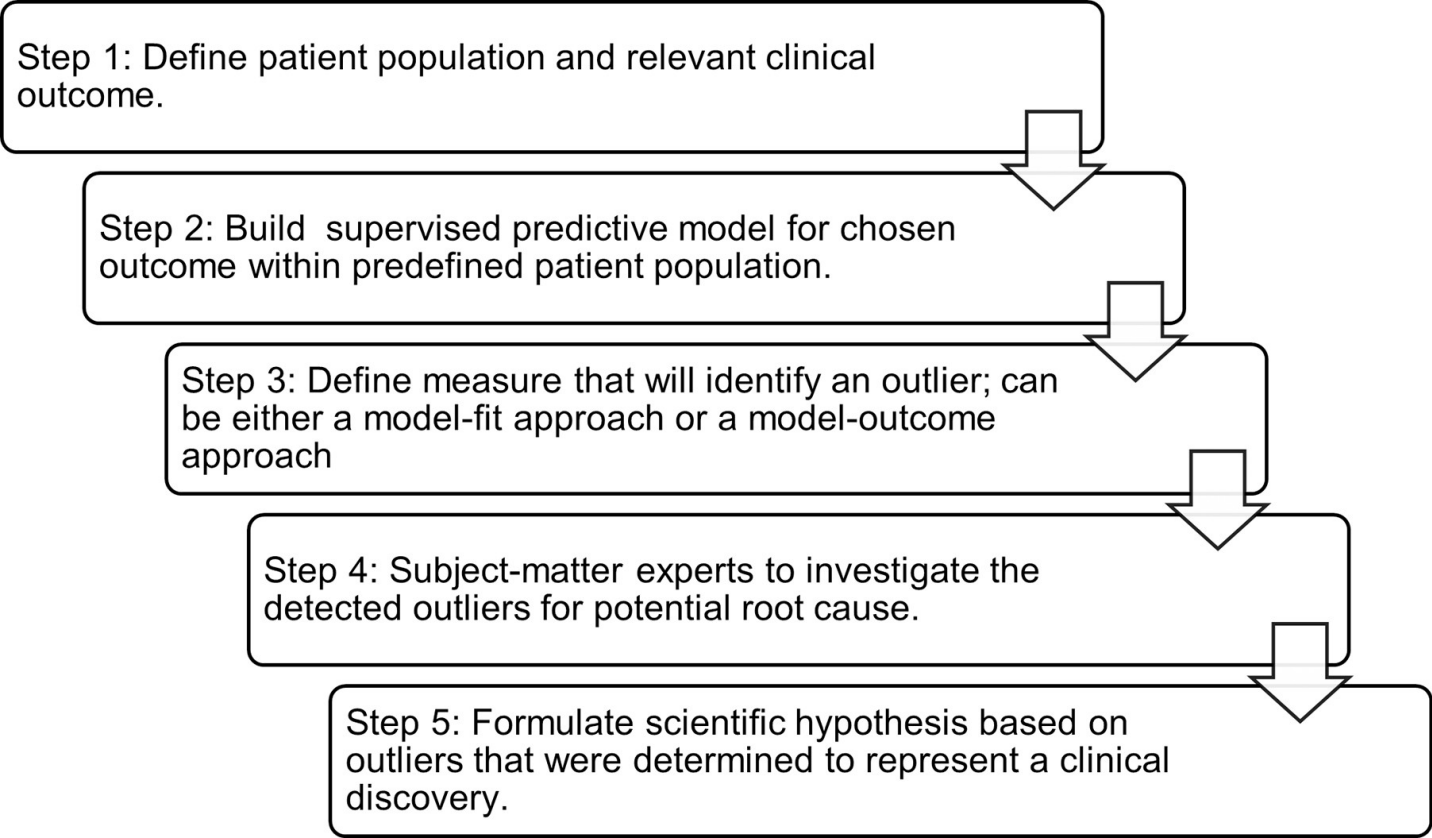
Overview of the Augmented Intelligence Framework for Accelerating Clinical Discovery Through Outlier Analysis.

We propose the following approach to structuring outlier analysis projects for clinical discovery. The aim of the following steps is to maximize the potential of capturing novelty-based outliers and to maintain consistency across projects. An overview of these steps can subsequently be found in Fig 2.

### Step 1: Define a patient population and a clinical outcome to be explored

Outlier analysis for clinical discovery falls within the paradigm of exploratory research. While it is possible to conduct unsupervised outlier analysis on any dataset to determine outlier observations, without setting (i.e., grounding on) the clinical context, the outlier output is unlikely to be informative for clinical discovery. Setting the clinical context starts with formulating two clinical parameters: population and outcome of interest. Additional parameters are possible (e.g., some exposure or intervention) but may lead to a reduction of the available data for analysis. Considering the rare event rate of clinical discovery observations, a large dataset is always desirable. This approach is similar to the population, intervention, comparison, and outcome (or “PICO”) framework for generating clinical questions within the paradigm of evidence-based medicine [74,75].

### Step 2: Build a supervised predictive model of the chosen patient population and clinical outcome

Various predictive modelling methods can be used to define the normal state and behaviour of the data in relation to the outcome. The resultant fit of the predictive model or its outcomes will serve as a quantitative basis to identify contextual outliers. Predictive models provide a feasible, reproducible, and objective approach to the definition of normal or expected behaviour within the dataset in question. A predictive model should be built and optimized using the population and outcome defined in Step 1. In its essence, the aim of a predictive model is two-fold: to summarize and reduce the multidimensionality of the observations and to allow the detection of contextual outliers rather than point outliers. Reducing multidimensional data into a low-dimensional subspace is a common approach in outlier analysis and is based on the assumption that outliers are masked by the full dimensionality of the data [71,76]. Applying a predictive model that attempts to predict an outcome of interest within a relevant patient population will ensure our ability to detect outliers within the context that is of interest. Detecting contextual outliers is a common challenge in outlier analysis, and one of the strategies to address this challenge is to reframe the analysis problem so as to only include the context of interest [71].

### Step 3: Determine the optimal measure to detect outliers

Next, we must determine what type of measure to use and the threshold of that measure that can distinguish an outlier observation from a normal observation. Using a predictive model approach, two main tactics can be used: model fit measures and model outcome measures.

In a model fit approach, an outlier would be an observation that, if removed, would result in a model that can better predict the outcome. Underlying this approach is the extent to which data heterogeneity affects model performance [77]. Depending on the predictive model used, there are a variety of model fit and model error measures that can be utilized. Torr and Murray (1993) utilize the iterative pruning of outliers and refitting of the model, aiming to minimize the least squares measure in a linear regression model [78]. In a similar fashion, John (1995) utilizes repeated pruning of decision-tree models until optimal tree representation is achieved. Nodes in the decision tree that were pruned out represent outliers and are systematically removed until a point where the majority of remaining points only represent normal points [79]. Also using a model fit approach, Hawkins and colleagues (2002) and Williams and colleagues (2002) utilize a replicator neural network, whereby each data instance is reconstructed using a learned model and the reconstruction error is directly used as an outlier score for each instance reconstructed [80,81]. These are a few examples of established approaches to leveraging model fit to determine outliers.

In a model outcome approach, an investigator would use the prediction model output as a basis for determining outlier observation. One intuitive approach is to rank misclassified observations (i.e., observations that the model predicts wrongly) based on the confidence the model has assigned to the wrong prediction. An observation that was wrongly predicted with high confidence can be considered an outlier, as it has deviated substantially from the normal expected behaviour. This approach is analogous to how clinicians are likely to think of an unusual clinical encounter, whereby a certain expected clinical outcome (e.g., an improvement or deterioration of a condition) is not achieved despite high confidence that it should have materialized. However, using the model confidence score of wrong predictions requires the use of a predictive model that provides such label scores (e.g., regression models). A similar approach to outlier analysis can be seen in Roberts and Tarassenko (1994); the authors used expectation maximization to estimate a model distribution and then proceeded to estimate the probability that a given observation was at the extreme value of the distribution [82]. A model outcome approach is a form of an information-based outlier measurement, as the removal of the misclassified observation would improve the overall accuracy of the model. However, using the model outcomes to assess outliers allows us to incorporate any outlier measure approach. This is possible because using the outcomes of a predictive model has effectively turned the original multidimensional and general dataset into a single-dimensional problem. Moreover, the predictive model has reframed the original dataset into a proper context in relation to the prediction model outcome. We suggest using the information-based outlier measurement approach through identifying misclassification, together with the degree of the model confidence in the predicted classification. This approach would allow the investigator to use virtually any outlier analysis measurement approach, as the task has now been turned into an analysis of univariant data to detect point outliers.

The determination of both the outlier measure and the threshold for classifying outliers can be influenced by the predictive model diagnostic. Given a threshold, a model with higher accuracy will likely produce fewer outliers than a less accurate one. However, the proportion of outliers with novelty as a root cause is likely higher in more accurate models than in less accurate models.

### Step 4: Investigate identified outlier observations for potential root causes

The general aim of outlier analysis is to understand what caused an observation to deviate from the expected or normal behaviour, outcome, or model. Specifically, it aims to identify those observations that have deviated from the norm due to a unique underlying mechanism that can advance our clinical understanding of the area under investigation. Once an outlier observation has been identified in a given dataset, a panel of content-matter experts should review all details associated with the identified observation to understand why it deviated from the norm. The panel of content-matter experts can attribute an outlier observation to one of the four outlier root causes described earlier: error (e.g., a data entry error), fault (e.g., faulty instruments, fraud), natural deviation, or novelty (i.e., clinical discovery). The use of expert knowledge in various stages of outlier analysis, including verification of the correctness and studying of outliers, has been reported in several places across the outlier analysis literature [83–85].

It is ideal that the panel of content-matter experts, once they identify a potential clinical discovery, seek additional information outside of what is captured in the existing data. The assumption here is that the underlying mechanism that gave rise to the outlier is not captured by the existing collected information in the dataset. Had such information been well-represented in the dataset, it is likely to have been accounted for in the predictive model.

### Step 5: Use outliers determined as clinical discovery to formulate scientific hypotheses

Outlier observations that have been concluded to represent a clinical discovery by the domain content-matter experts panel should be studied, and a scientific hypothesis should be generated from these observations. It is important to note that the output of this framework is exploratory in nature and cannot provide any type of statistical inference, including causal inference. Any clinical insight gained from this framework must be further assessed in a proper comparative study design. The aim of this augmented intelligence framework is to accelerate the rate of clinical discovery through the use of outlier analysis as opposed to relying on the classic approach to clinical discovery that is largely human-based. The increased efficiency will likely provide a greater rate of novel and promising insights.

## Systematic review of outlier analysis in obstetric research

Due to the challenges of conducting clinical trials with pregnant participants, obstetrics research is not supported by a strong industry that is incentivized to accelerate and commercialize discoveries. We are of the opinion that the field of obstetrics would benefit from applying outlier analysis to accelerate clinical discovery. To explore and assess the use of outlier analysis in obstetrics research, we conducted a systematic review.

### Methods

#### Search strategy

In consultation with a medical information specialist, we developed two search strategies covering three bibliographic databases. We provide the detailed search strategies in S1 Appendix. We included controlled vocabulary, as well as keywords, with main search concepts related to obstetrics and outlier analysis methods. One search strategy was developed to search Embase and MEDLINE through the Ovid search engine. The second search strategy was developed to search Web of Science. We conducted the search on September 2, 2021.

#### Study selection

We list studies that met our eligibility criteria in Table 2. Studies had to include a population of pregnant women and utilize an outlier analysis method that aimed to identify unusual observations rather than to remove statistical noise. We defined an outlier analysis method as any approach that has both of the two following elements:

- establishes or defines the normal or expected behaviour of the data or population being analyzed
- identifies specific observations or patterns in the data that do not conform to the established normal or expected behaviour.

**Table 2:** Eligibility Criteria for the Systematic Review.

	Eligibility Criteria
Population	Studies with a population that includes a pregnant person
Intervention/ Exposure	Any
Comparators	Not applicable
Outcomes	Any
Study Design	Any
Study Methods	The use of outlier analysis as part of the methods. Outlier analysis methods include any approach that: • establishes or defines a normal or expected behaviour, outcome, or model of the data or population being analyzed • identifies specific observations or patterns in the data that do not conform to the established norm or expectation
Other	• English language • Full text available

These two criteria apply well to the definition of outliers discussed earlier and do not restrict the outlier approach to specific published statistical models and algorithms. These criteria would further allow the inclusion of non-quantitative approaches, such as a clinical decision rule-based approach to outlier analysis.

Two reviewers (GJ and MU) independently screened the title and abstract of the results retrieved from the search. Subsequently, we screened the full text of the eligible abstracts for the inclusion and exclusion criteria. We solved any disagreement between the two reviewers through discussion, and if we were not able to reach an agreement, a third independent reviewer (MW) provided arbitration.

#### Data extraction and synthesis

One reviewer (GJ) extracted data relevant to the following categories: study characteristics, population characteristics, intervention/exposure, outcome measures, and outlier analysis method. Study characteristics included study design, publication year, setting, country of origin, inclusion and exclusion criteria, and sample size. Population characteristics included population selection criteria, baseline demographics, and other patient characteristics. Intervention/exposure included the type, characteristics, and duration of the intervention. Outcome measures included the definition and results. Outlier analysis included the type, features, approach to feature engineering, approach to data sampling for training-based models, model diagnostics and optimization, model performance results, and model validation.

To ensure accurate data extraction, a second reviewer (MU) conducted a check of the accuracy of 20% of the extracted data. As the aim of this systematic review was to explore and assess the use of outlier analysis methods, we did not conduct a quantitative meta-analysis for this systematic review. Instead, we planned an a priori narrative synthesis of the included studies.

#### Quality assessment

Currently, there is no standardized quality assessment tool for outlier analysis studies in clinical research. However, for those studies that included a predictive component, we used the *Critical Appraisal and Data Extraction for Systematic Reviews of Prediction Modelling Studies*: *The CHARMS Checklist* [86].

### Results

**Fig 3:**
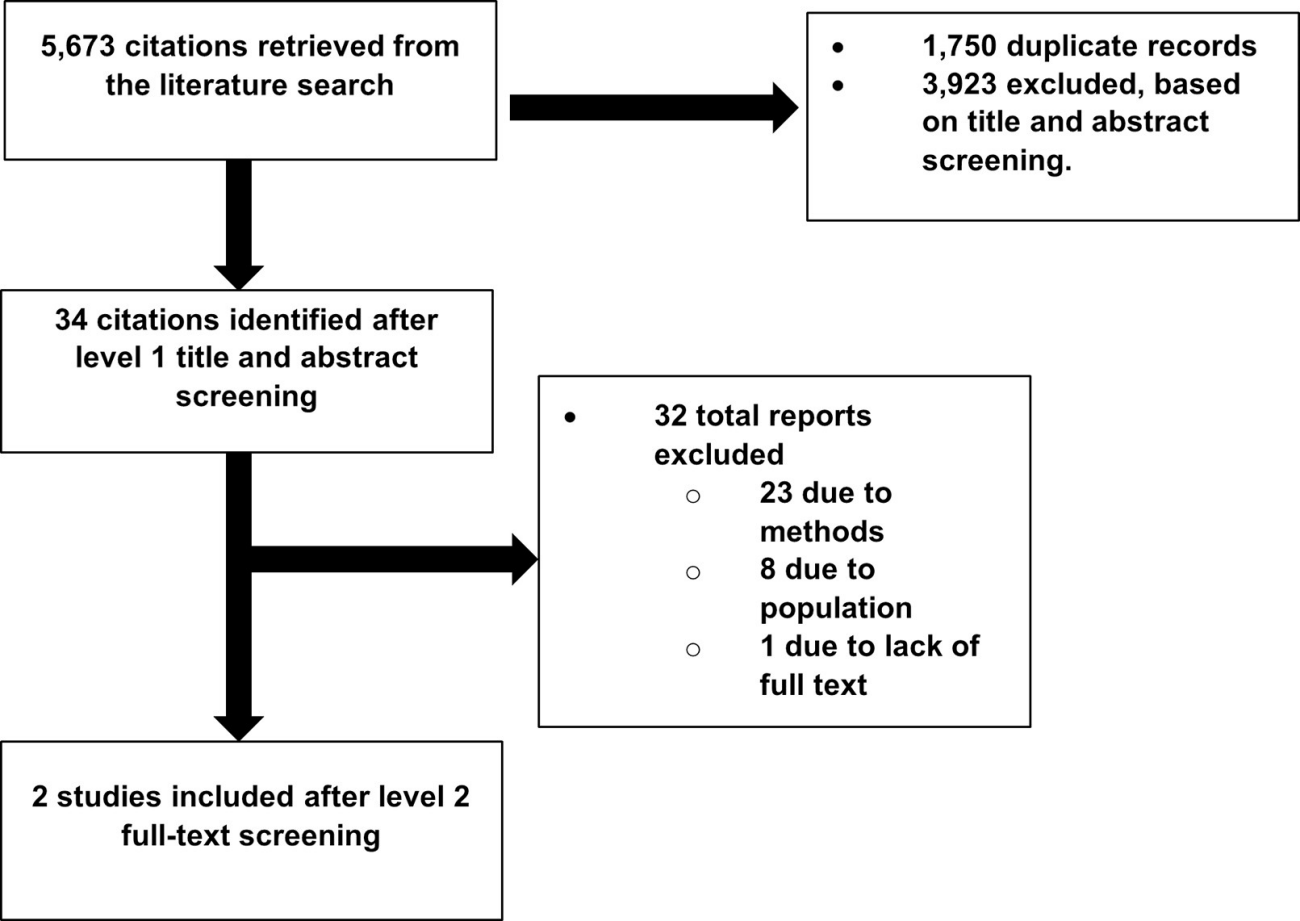
PRISMA Flow Chart of Included and Excluded Studies.

We retrieved 5,673 records after running our search. Subsequent to title and abstract screening, we selected 34 records for full-text screening. Of these, we further excluded 32 reports and found that two reports representing two unique studies met our inclusion and exclusion criteria [87,88]. A list of all excluded reports, together with the reason for exclusion, can be found in S2 Appendix. A PRISMA flow chart for included and excluded studies can be found in Fig 3.

We did not assess the quality of either included study, as neither one had a predictive component.

One of the included studies, Antonelli and colleagues proposed a framework for detecting clinical practice anomalies in health-related databases. The proposed framework consisted of determining patterns in medical records and comparing these patterns to published medical guidelines. Antonelli and colleagues (2013) applied the proposed framework to patient data from 905 pregnant persons. The authors analyzed the data for patterns in the frequencies in which the pregnant persons visited their healthcare providers for routine antenatal visits and contrasted these patterns with the medical guidelines from the Italian Ministry of Health (Ministerial Decree, 1998). Antonelli and colleagues did not describe a quantitative method for defining or identifying anomalous patterns. Instead, the authors considered any antenatal visit pattern that did not adhere to the medical guidelines as an anomaly [87].

The main two anomaly patterns reported by Antonelli and colleagues (2013) were the lack of fully utilizing the free examinations offered by the Italian Ministry of Health and the higher frequency of examinations during the second and the third trimesters than recommended by the guidelines [87]. The second included study by Khan and colleagues (2017)^78^ aimed, among other objectives, to identify spatial outliers of teenage birth rate in counties in the US. The authors utilized the National Vital Statistics System Birth Data files between 2003 and 2012 to provide a count of teen births at a county level. To identify counties with an outlier teen birth rate, the authors used Anselin Local Moran’s I cluster and outlier analysis method to examine spatial outliers, with positive non-zero weights assigned to the eight closest neighbours to the target county. Spatial outliers were counties with a low or high teen birth rate, surrounded by counties with a high or low teen birth rate, respectively [88].

Khan and colleagues (2017) identified a total of 44 outliers in 2003: 30 counties had a high teen birth rate surrounded by counties with a low teen birth rate, and 14 counties had a low teen birth rate surrounded by counties with a high teen birth rate. In 2012, Khan and colleagues identified 40 outliers: 24 counties had a high teen birth rate surrounded by a low teen birth rate, and 16 counties had a low teen birth rate surrounded by a high birth rate [88].

A summary of the findings from both studies is presented in Table 3.

**Table 3:** Summary of findings.

Study Details	Antonelli et al. (2013)	Khan et al. (2017)
**Objective**	Detect clinical practice anomalies in health-related databases	Identify spatial outliers of teenage birth rate in US counties
**Framework/Methodology**	Patterns in medical records compared to published medical guidelines. Anomalies were identified when patterns did not adhere to guidelines.	Anselin Local Moran’s I cluster and outlier analysis method. Spatial outliers were identified based on high or low teen birth rates contrasted with surrounding counties’ rates.
**Data Source**	Patient data from 905 pregnant persons	National Vital Statistics System Birth Data files between 2003–2012
**Primary Guidelines/Data Reference**	Italian Ministry of Health (Ministerial Decree, 1998) for antenatal visits	Teen births at a county level
**Main Findings**	Two main anomaly patterns: 1) Underutilization of free examinations. 2) Higher frequency of examinations in the 2nd and 3rd trimesters than recommended.	44 outliers in 2003: 30 counties with high birth rates contrasted by surrounding low rates, 14 with low rates contrasted by surrounding high rates. In 2012, 24 counties with high birth rates contrasted by surrounding low rates, 16 the other way around.
**Method of Identifying Anomalies/Outliers**	Any antenatal visit pattern differing from the medical guidelines	Spatial outliers using positive non-zero weights assigned to the eight closest neighbours to the target county.
**Total Anomalies/Outliers Reported**	Not Quantitatively Described	44 outliers in 2003 and 40 outliers in 2012
**Outliers type**	Point outliers	Point outliers
**Outliers measure**	Information	Distance
**Outliers root cause**	Unclear	Unclear

## Discussion

The philosophy of science has long grappled with the concept of scientific discovery and the methodologies leading to such insights. Prominent 17th-century thinkers, including Bacon, Descartes, and Newton, posited that specific methods of inquiry would lead not just to discoveries but also to unearthing definitive intellectual truths [89]. However, the 19th century witnessed a wane in these conventional inquiry methods, attributed to influences like Romanticism and the inadequacy of prior models to propel scientific progress. This evolution, coupled with advancements in mathematical and statistical techniques, birthed the now-prevailing hypothetico-deductive model, which prioritizes the testing of falsifiable hypotheses over their genesis [89].

Clinical research predominantly adheres to the hypothetico-deductive model, as evidenced by the widespread adoption of the null and alternative hypothesis in comparative clinical research and the elevated status of randomized controlled trials as the gold standard [90]. Yet, clinical research distinguishes itself with the tenet of clinical equipoise, which mandates the justification of the rationale and beliefs underpinning a hypothesis [91].

Notably, clinical research isn’t the sole field that underscores the genesis of a hypothesis. Data-driven discovery, a method frequently employed in domains like genomics and astronomy, emphasizes gleaning insights straight from vast datasets. Standing in contrast to traditional hypothesis-driven methods, this approach gives precedence to the data itself as the foundational basis [92–95].

Increasing the rate of clinical discoveries will inevitably lead to better therapeutics, diagnostics, and patient care. The existing practices in identifying, investigating, and communicating unusual clinical observations through case reports and case studies are inefficient, resource-intensive, and do not utilize existing technologies. In this article, we proposed a framework for using patient data, applying outlier analysis, and investigating outliers to accelerate the rate of clinical discoveries. We have presented a non-technical introduction to outlier analysis, with applicable clinical examples, to allow for an intuitive understanding of the process by healthcare professionals.

The use of outlier analysis methods to detect suspicious data and abnormal cases for further clinical investigation may have been first suggested in a publication in the year 2000 by Laurikkala and colleagues [84]. We were unable to find further published literature that suggests using outlier analysis with the input of content-matter experts to uncover novel clinical observations.

The use of various data analytics approaches to support or augment a traditional human process is a cornerstone of augmented intelligence and symbiotic autonomous systems [96]. As stated by Broschert and colleagues (2019), part of the promised applications of augmented intelligence is the “medical analysis of case files to identify efficient treatment options.” [96] We provided here a detailed, step-by-step process on how to utilize generic statistical and machine-learning approaches in augmenting all aspects of clinical discovery. We built our framework explicitly to simulate the classic clinical and bedside process of discovery that has already contributed immeasurably to medicine. This framework has the potential to rapidly accelerate the classic clinical discovery process. We envision that this framework could be used with both existing data from clinical studies as well as live data from patient records or ongoing clinical trials. A research unit could be designated to build and maintain the model and the outlier analysis measure, while a committee of domain experts could continuously investigate identified outliers. This process could run perpetually and generate continuous insights for both quality assurance and control of the data, as well as for identifying promising areas of study to be investigated. In addition, such a framework would reduce human bias toward pursuing certain unusual clinical observations over others. Furthermore, the structured approach of this framework opens the opportunity for collaboration and synthesis of clinical discovery across various groups, as each of the steps outlined can be replicated and measured.

Recognizing the potential of accelerating clinical discoveries in obstetrics—a field that has been traditionally neglected by the pharmaceutical industry—we conducted a systematic review to explore the existing use of outlier analysis in obstetric research. Our systematic review identified two obstetrics-related studies that utilized outlier analysis for the purposes of outlier detection. Both studies assessed outliers at an aggregate level rather than at an individual patient level. Neither study utilized a predictive model to represent a normal behaviour for which an observation is contrasted to determine its outlier status. Instead, one study used clinical guidelines as the expected normal behaviour, while the other study used the k-nearest neighbours algorithm approach—a particular outlier analysis approach that is best suited for graphical data as opposed to multidimensional data (e.g., patient data).

To our knowledge, this is the first systematic review of outlier detection studies in obstetric research. In 2011, Gaspar and colleagues performed a systematic review of outlier detection techniques in medical administrative data. Gaspar and colleagues identified 177 papers for inclusion but reported on 80 randomly selected papers. The authors’ findings suggest that the majority of the reported papers were in the fields of oncology (32%), quality indicators (24%), genetics (15%), and neurology (12%) [39]. The primary papers in Gaspar et al. indicate that outlier analysis has been successfully used to identify gene targets in prostate cancer, [97] drive insights as to the reasons behind long hospital admissions in patients with heart failure, [98] and improve data quality in medical registries [99]. Outlier analysis is sometimes discussed under the topic of data mining, whereby the aim of the discipline is to uncover useful information and knowledge that is hidden in large amounts of data [100]. Data mining has been widely used as an exploratory analysis tool to uncover hidden associations in clinical databases [101–109].

Limitations to our systematic review include the restriction of the search strategy to the English language, which may have missed publications in other languages. Another limitation is the lack of standardized definitions and the interdisciplinary nature of outlier analysis methods. This lack of standard terminology and the large number of disciplines utilizing outlier analysis with potentially different terminology may have hindered our search strategy and screening efforts, whereby terminology that did not conform to the reviewers’ expectations may have been missed. Finally, there is no standard method to assess the quality of outlier studies. This limited our ability to conduct an assessment of the quality of the included studies.

In a paper published after the systematic review’s search date, we applied the Augmented Intelligence framework to data on hypertensive disorders of pregnancy from the FACT randomized controlled trial (N = 2,301) and the OaK prospective cohort study (N = 8,085). Using a random forest predictive model, we predicted preeclampsia and other hypertensive disorders, marking those misclassified with over 90% confidence as outliers. This method, termed extreme misclassification contextual outlier (EMCO) analysis, was compared to the traditional isolation forest outlier method. Out of the 302 outliers, clinical experts identified 49 as representing potential novelties. The EMCO method pinpointed 111 (1.1%) outliers, in contrast to the 191 (1.8%) from the isolation forest. Notably, EMCO identified a higher proportion of potential novelties (37.8%) than the isolation forest (3.7%). Within the EMCO method, the FACT study model outperformed the OaK model. While OaK had more outliers at 98 (1.2%) compared to FACT’s 13 (0.6%), FACT had a higher rate of potential novelties (76.9%) than OaK (32.7%) [70].

## Conclusion

Outlier analysis can be utilized to fuel the classic process of clinical discovery in an augmented intelligence framework. The classic approach to clinical discovery has been largely human-based. Our augmented intelligence framework provides a structured and multidisciplinary approach that can be implemented with any patient data. The aim of our framework is to accelerate the rate of clinical discovery through the use of outlier analysis. The increased efficiency will likely provide a greater rate of novel and promising insights. The field of obstetrics can particularly benefit from implementing this framework, given the chronic exclusion of pregnant individuals from biopharmaceutical studies, but methods for application in obstetrics research currently require ongoing development.

## Supporting information

S1 Appendix Search Strategy. (DOCX)

S2 Appendix List of Excluded Studies With Reason—Post Full-Text Screening. (DOCX)
