# Supplementary material for: Outlier analysis for accelerating clinical discovery: An augmented intelligence framework and a systematic review
Source: PLOS Digit Health. 2024 May 22;3(5):e0000515. doi: 10.1371/journal.pdig.0000515 (PMC11111092; doi:10.1371/journal.pdig.0000515)
Supplement: S2 Appendix — (DOCX) [file pdig.0000515.s002.docx]

# S2 Appendix – List of Excluded Studies With Reason ― Post Full-Text Screening

| Study | Reason for Exclusion |
| --- | --- |
| Allotey PA and Harel O (2019). "Multiple Imputation for Incomplete Data in Environmental Epidemiology Research."[1] | Methods do not match the required eligibility criteria. |
| Bate A (2007). "Bayesian Confidence Propagation Neural Network."[2] | Methods do not match the required eligibility criteria. |
| Bengtson AM et al. (2016). "Multiple Overimputation to Address Missing Data and Measurement Error: Application to HIV Treatment During Pregnancy and Pregnancy Outcomes."[3] | Methods do not match the required eligibility criteria. |
| Boland MR et al. (2017). "Development of A Machine Learning Algorithm to Classify Drugs of Unknown Fetal Effect."[4] | Methods do not match the required eligibility criteria. |
| Cardoso-dos-Santos AC et al. (2018). "Twin Peaks: A Spatial and Temporal Study of Twinning Rates in Brazil."[5] | Methods do not match the required eligibility criteria. |
| Chauvet PE et al. (2014). "Evaluation of Automatic Feature Detection Algorithms in EEG: Application to Interburst Intervals."[6] | Methods do not match the required eligibility criteria. |
| Chen HC et al. (2013). "Data Mining for Signal Detection of Adverse Event Safety Data."[7] | Population does not match the required eligibility criteria. |
| Ferguson KK et al. (2018). "Foetal Ultrasound Measurement Imputations Based on Growth Curves Versus Multiple Imputation Chained Equation (MICE)."[8] | Methods do not match the required eligibility criteria. |
| Feyaerts D et al. (2018). "Endometrial Natural Killer (NK) Cells Reveal a Tissue-Specific Receptor Repertoire."[9] | Methods do not match the required eligibility criteria. |
| Giezen TJ et al. (2010). "Mapping the Safety Profile of Biologicals: A Disproportionality Analysis Using the Who Adverse Drug Reaction Database, Vigibase."[10] | Methods do not match the required eligibility criteria. |
| Harel O et al. (2018). "Multiple Imputation for Incomplete Data in Epidemiologic Studies."[11] | Methods do not match the required eligibility criteria. |
| Karayiannis NB et al. (2006). "Automated Detection of Videotaped Neonatal Seizures Based on Motion Tracking Methods."[12] | Population does not match the required eligibility criteria. |
| Lyles RH and Allen AS (2002). "Estimating Crude or Common Odds Ratios in Case-Control Studies with Informatively Missing Exposure Data."[13] | Methods do not match the required eligibility criteria. |
| Menon R et al. (2014). "Multivariate Adaptive Regression Splines Analysis to Predict Biomarkers of Spontaneous Preterm Birth."[14] | Methods do not match the required eligibility criteria. |
| Noto K et al. (2015). "CSAX: Characterizing Systematic Anomalies in eXpression Data."[15] | Population does not match the required eligibility criteria. |
| Padmavathy S and Suresh P (2019). "Fetal Ultrasound Image Evaluation of Chromosomal Anomaly Detection and Classification Using Conditional Rooted Neural Network (CRNN)."[16] | No full text was obtainable. |
| Perkins NJ et al. (2018). "Principled Approaches to Missing Data in Epidemiologic Studies."[17] | Methods do not match the required eligibility criteria. |
| Sairanen V et al. (2018). "Fast and Accurate Slicewise OutLIer Detection (SOLID) With Informed Model Estimation for Diffusion MRI Data."[18] | Population does not match the required eligibility criteria. |
| Sbrollini A et al. (2018). "eCTG: An Automatic Procedure to Extract Digital Cardiotocographic Signals from Digital Images."[19] | Methods do not match the required eligibility criteria. |
| Stevens J et al. (2014). "Effects of Changes in Click-Evoked Otoacoustic Emission (CEOAE) Pass Criteria, As Used in the English Newborn Hearing Screening Program, on Screening Outcome."[20] | Methods do not match the required eligibility criteria. |
| Sun H et al. (2019). "Identification of Suspicious Invasive Placentation Based on Clinical MRI Data Using Textural Features and Automated Machine Learning."[21] | Methods do not match the required eligibility criteria. |
| Temko A and Lightbody G (2016). "Detecting Neonatal Seizures with Computer Algorithms."[22] | Population does not match the required eligibility criteria. |
| Temko A et al. (2011). "EEG-Based Neonatal Seizure Detection with Support Vector Machines."[23] | Population does not match the required eligibility criteria. |
| Temko A et al. (2011). "Performance Assessment For EEG-Based Neonatal Seizure Detectors."[24] | Population does not match the required eligibility criteria. |
| Tilling K et al. (2016). "Appropriate Inclusion of Interactions Was Needed to Avoid Bias in Multiple Imputation."[25] | Methods do not match the required eligibility criteria. |
| Turnbull JP et al. (2001). "Automated Detection of Trace Alternant During Sleep in Healthy Full-Term Neonates Using Discrete Wavelet Transform."[26] | Population does not match the required eligibility criteria. |
| Varanini M et al. (2017). "A New Method for QRS Complex Detection in Multichannel ECG: Application to Self-Monitoring of Fetal Health."[27] | Methods do not match the required eligibility criteria. |
| Wang WL and Lin TI (2014). "Multivariate T Nonlinear Mixed-Effects Models for Multi-Outcome Longitudinal Data With Missing Values."[28] | Methods do not match the required eligibility criteria. |
| Wright DB and London K (2009). "Multilevel Modelling: Beyond the Basic Applications."[29] | Methods do not match the required eligibility criteria. |
| Yu Q et al. (2018). "Automatic Identifying of Maternal ECG Source When Applying ICA in Fetal ECG Extraction."[30] | Methods do not match the required eligibility criteria. |
| Zhao LP et al. (1996). "Regression Analysis with Missing Covariate Data Using Estimating Equations."[31] | Methods do not match the required eligibility criteria. |
| Ziegler A et al. (2003). "Analysis of Pregnancy and Other Factors on Detection of Human Papilloma Virus (HPV) Infection Using Weighted Estimating Equations for Follow-Up Data."[32] | Methods do not match the required eligibility criteria. |

# References

1. Allotey PA, Harel O. Multiple Imputation for Incomplete Data in Environmental Epidemiology Research. Current environmental health reports. 2019;6(2):62-71. doi: <http://dx.doi.org/10.1007/s40572-019-00230-y>. PubMed PMID: 627865432.

2. Bate A. Bayesian confidence propagation neural network. Drug Saf. 2007;30(7):623-5. PubMed PMID: 17604417.

3. Bengtson AM, Westreich D, Musonda P, Pettifor A, Chibwesha C, Chi BH, et al. Multiple Overimputation to Address Missing Data and Measurement Error: Application to HIV Treatment During Pregnancy and Pregnancy Outcomes. Epidemiology. 2016;27(5):642-50. doi: <https://dx.doi.org/10.1097/EDE.0000000000000494>. PubMed PMID: 27054651.

4. Boland MR, Polubriaginof F, Tatonetti NP. Development of A Machine Learning Algorithm to Classify Drugs Of Unknown Fetal Effect. Scientific reports. 2017;7(1):12839. doi: <http://dx.doi.org/10.1038/s41598-017-12943-x>. PubMed PMID: 624329898.

5. Cardoso-dos-Santos AC, Boquett J, Zagonel de Oliveira M, Callegari-Jacques SM, Barbian MH, Sanseverino MTV, et al. Twin Peaks: A spatial and temporal study of twinning rates in Brazil. PLoS ONE. 2018;13 (7) (no pagination)(e0200885). doi: <http://dx.doi.org/10.1371/journal.pone.0200885>. PubMed PMID: 623114871.

6. Chauvet PE, Tich SNT, Schang D, Clement A. Evaluation of automatic feature detection algorithms in EEG: Application to interburst intervals. Computers in Biology and Medicine. 2014;54:61-71. doi: <http://dx.doi.org/10.1016/j.compbiomed.2014.08.011>. PubMed PMID: 600258972.

7. Chen HC, Tsong Y, Chen JJ. Data mining for signal detection of adverse event safety data. Journal of Biopharmaceutical Statistics. 2013;23(1):146-60. doi: <http://dx.doi.org/10.1080/10543406.2013.735780>. PubMed PMID: 368258343.

8. Ferguson KK, Yu Y, Cantonwine DE, McElrath TF, Meeker JD, Mukherjee B. Foetal ultrasound measurement imputations based on growth curves versus multiple imputation chained equation (MICE). Paediatric and Perinatal Epidemiology. 2018;32(5):469-73. doi: <http://dx.doi.org/10.1111/ppe.12486>. PubMed PMID: 623247851.

9. Feyaerts D, Kuret T, Van Cranenbroek B, Van Der Zeeuw-Hingrez S, Van Der Heijden OWH, Van Der Meer A, et al. Endometrial natural killer (NK) cells reveal a tissue-specific receptor repertoire. Human Reproduction. 2018;33(3):441-51. doi: <http://dx.doi.org/10.1093/humrep/dey001>. PubMed PMID: 621082209.

10. Giezen TJ, Mantel-Teeuwisse AK, Meyboom RHB, Straus SMJM, Leufkens HGM, Egberts TCG. Mapping the safety profile of biologicals: A disproportionality analysis using the who adverse drug reaction database, vigibase. Drug Saf. 2010;33(10):865-78. doi: <http://dx.doi.org/10.2165/11538330-000000000-00000>. PubMed PMID: 359477540.

11. Harel O, Mitchell EM, Perkins NJ, Cole SR, Tchetgen Tchetgen EJ, Sun B, et al. Multiple Imputation for Incomplete Data in Epidemiologic Studies. American Journal of Epidemiology. 2018;187(3):576-84. doi: <http://dx.doi.org/10.1093/aje/kwx349>.

12. Karayiannis NB, Xiong Y, Frost Jr JD, Wise MS, Hrachovy RA, Mizrahi EM. Automated detection of videotaped neonatal seizures based on motion tracking methods. Journal of Clinical Neurophysiology. 2006;23(6):521-31. doi: <http://dx.doi.org/10.1097/00004691-200612000-00004>. PubMed PMID: 44901059.

13. Lyles RH, Allen AS. Estimating crude or common odds ratios in case-control studies with informatively missing exposure data. American Journal of Epidemiology. 2002;155(3):274-81. PubMed PMID: 11821253.

14. Menon R, Bhat G, Saade GR, Spratt H. Multivariate adaptive regression splines analysis to predict biomarkers of spontaneous preterm birth. Acta Obstetricia et Gynecologica Scandinavica. 2014;93(4):382-91. doi: <http://dx.doi.org/10.1111/aogs.12344>. PubMed PMID: 372699226.

15. Noto K, Majidi S, Edlow AG, Wick HC, Bianchi DW, Slonim DK. CSAX: Characterizing Systematic Anomalies in eXpression Data. Journal of computational biology : a journal of computational molecular cell biology. 2015;22(5):402-13. doi: <http://dx.doi.org/10.1089/cmb.2014.0155>. PubMed PMID: 615351220.

16. Padmavathy S, Suresh P. Fetal ultrasound image evaluation of chromosomal anomaly detection and classification using Conditional Rooted neural network (CRNN). Journal of Medical Imaging and Health Informatics. 2019;9(6):1307-15. doi: <http://dx.doi.org/10.1166/jmihi.2019.2734>. PubMed PMID: 2002084150.

17. Perkins NJ, Cole SR, Harel O, Tchetgen Tchetgen EJ, Sun B, Mitchell EM, et al. Principled Approaches to Missing Data in Epidemiologic Studies. American Journal of Epidemiology. 2018;187(3):568-75. doi: <http://dx.doi.org/10.1093/aje/kwx348>. PubMed PMID: 621080337.

18. Sairanen V, Leemans A, Tax CMW. Fast and accurate Slicewise OutLIer Detection (SOLID) with informed model estimation for diffusion MRI data. NeuroImage. 2018;181:331-46. doi: <http://dx.doi.org/10.1016/j.neuroimage.2018.07.003>. PubMed PMID: 2000954359.

19. Sbrollini A, Agostinelli A, Marcantoni I, Morettini M, Di Nardo F, Fioretti S, et al. eCTG: an automatic procedure to extract digital cardiotocographic signals from digital images. Computer Methods and Programs in Biomedicine. 2018;156:133-9. doi: <http://dx.doi.org/10.1016/j.cmpb.2017.12.030>. PubMed PMID: 620762859.

20. Stevens J, Brandreth M, Bacon P. Effects of changes in click-evoked otoacoustic emission (CEOAE) pass criteria, as used in the English newborn hearing screening program, on screening outcome. Int J Audiol. 2014;53(9):613-7. doi: <https://dx.doi.org/10.3109/14992027.2014.905715>. PubMed PMID: 24825366.

21. Sun H, Qu H, Chen L, Wang W, Liao Y, Zou L, et al. Identification of suspicious invasive placentation based on clinical MRI data using textural features and automated machine learning. European Radiology. 2019;29(11):6152-62. doi: <http://dx.doi.org/10.1007/s00330-019-06372-9>. PubMed PMID: 2002667081.

22. Temko A, Lightbody G. Detecting neonatal seizures with computer algorithms. Journal of Clinical Neurophysiology. 2016;33(5):394-402. doi: <http://dx.doi.org/10.1097/WNP.0000000000000295>. PubMed PMID: 612541095.

23. Temko A, Thomas E, Marnane W, Lightbody G, Boylan G. EEG-based neonatal seizure detection with Support Vector Machines. Clinical Neurophysiology. 2011;122(3):464-73. doi: <http://dx.doi.org/10.1016/j.clinph.2010.06.034>. PubMed PMID: 51033110.

24. Temko A, Thomas E, Marnane W, Lightbody G, Boylan GB. Performance assessment for EEG-based neonatal seizure detectors. Clinical Neurophysiology. 2011;122(3):474-82. doi: <http://dx.doi.org/10.1016/j.clinph.2010.06.035>. PubMed PMID: 51033295.

25. Tilling K, Williamson EJ, Spratt M, Sterne JAC, Carpenter JR. Appropriate inclusion of interactions was needed to avoid bias in multiple imputation. Journal of Clinical Epidemiology. 2016;80:107-15. doi: <http://dx.doi.org/10.1016/j.jclinepi.2016.07.004>. PubMed PMID: 613223546.

26. Turnbull JP, Loparo KA, Johnson MW, Scher MS. Automated detection of trace alternant during sleep in healthy full-term neonates using discrete wavelet transform. Clinical Neurophysiology. 2001;112(10):1893-900. doi: <http://dx.doi.org/10.1016/S1388-2457%2801%2900641-1>. PubMed PMID: 32831529.

27. Varanini M, Tartarisco G, Balocchi R, Macerata A, Pioggia G, Billeci L. A new method for QRS complex detection in multichannel ECG: Application to self-monitoring of fetal health. Computers in Biology and Medicine. 2017;85:125-34. doi: <http://dx.doi.org/10.1016/j.compbiomed.2016.04.008>. PubMed PMID: 609991860.

28. Wang WL, Lin TI. Multivariate t nonlinear mixed-effects models for multi-outcome longitudinal data with missing values. Statistics in Medicine. 2014;33(17):3029-46. doi: <http://dx.doi.org/10.1002/sim.6144>. PubMed PMID: 53061584.

29. Wright DB, London K. Multilevel modelling: Beyond the basic applications. The British journal of mathematical and statistical psychology. 2009;62(Pt 2):439-56. doi: <http://dx.doi.org/10.1348/000711008X327632>. PubMed PMID: 355008542.

30. Yu Q, Yan H, Song L, Guo W, Liu H, Si J, et al. Automatic identifying of maternal ECG source when applying ICA in fetal ECG extraction. Biocybernetics and Biomedical Engineering. 2018;38(3):448-55. doi: <http://dx.doi.org/10.1016/j.bbe.2018.03.003>. PubMed PMID: 2000733199.

31. Zhao LP, Lipsitz S, Lew D. Regression analysis with missing covariate data using estimating equations. Biometrics. 1996;52(4):1165-82. PubMed PMID: 8962448.

32. Ziegler A, Kastner C, Chang-Claude J. Analysis of pregnancy and other factors on detection of human papilloma virus (HPV) infection using weighted estimating equations for follow-up data. Statistics in Medicine. 2003;22(13):2217-33. doi: <http://dx.doi.org/10.1002/sim.1409>. PubMed PMID: 36818046.
